# Complications of Poly-l-Lactic Acid and Polyglycolic Acid (PLLA/PGA) Osteosynthesis Systems for Maxillofacial Surgery: A Retrospective Clinical Investigation

**DOI:** 10.3390/polym13060889

**Published:** 2021-03-14

**Authors:** Yuhei Matsuda, Masaaki Karino, Tatsuo Okui, Takahiro Kanno

**Affiliations:** Department of Oral and Maxillofacial Surgery, Faculty of Medicine, Shimane University, Izumo, Shimane 693-8501, Japan; yuhei@med.shimane-u.ac.jp (Y.M.); karino71@med.shimane-u.ac.jp (M.K.); tokui@med.shimane-u.ac.jp (T.O.)

**Keywords:** poly-l-lactic acid and polyglycolic acid (PLLA/PGA), maxillofacial surgery, complication, osteosynthesis, retrospective, infra zygomatic crest, propensity score

## Abstract

Two second-generation PLLA/PGA bioresorbable osteosynthetic plate systems for oral and maxillofacial surgery are available in Japan. The two systems have different PLLA-PGA component ratios (RapidSorb^®^, 85:15; Lactosorb^®^, 82:18) and plate and screw shapes. We conducted a retrospective study to compare our clinical evaluation and examine the incidence of postoperative complications between the two plate systems. A retrospective survey was conducted in 148 patients (midfacial fracture/trauma (68.2%) and dentofacial deformity patients (31.8%); males (54.7%); median age, 37.5 years) treated using maxillofacial osteosynthetic plate systems. The complications included plate exposure (7.4%), infection, (2.7%), and plate breakage (0.7%). Multivariate logistic regression analysis showed a significant correlation between sex (female), plate system (Lactosorb^®^), number of plates, and pyriform aperture and periorbital sites of plate placement (*p* < 0.05). Additionally, the propensity score-adjusted model showed a significant correlation between Lactosorb^®^ and postoperative complications (odds ratio 1.007 (95% confidence interval, 1.001–1.055), *p* < 0.01). However, the two plate systems showed a low incidence rate of complications, and the plate integration and survivability were similar using 2.0-mm or 1.5-mm resorbable plate regardless of the plate system. Our findings suggest that female sex and a greater number of plates are risk factors for postoperative complications, whereas pyriform aperture and periorbital plate placements reduce the risk.

## 1. Introduction

Bioresorbable and biodegradable osteosynthetic fixation plate systems have been considered an effective fixation system that offers several advantages over titanium fixation, including the absence of corrosion and metal accumulation in tissues and the need to remove the implants after osseous healing [1].

For maxillofacial osteosynthesis, we used poly-l-lactic acid (PLLA) and poly-d-lactic acid (PDLA), the first-generation bioresorbable osteosynthetic poly materials [2]. This bioresorbable osteofixation implant material is free of toxic and mutagenic effects. However, some critical problems are related to using this first regenerative bioresorbable materials, such as an inflammatory response, higher refracture rates, insufficient mechanical properties/support, foreign-body reactions, a late-degradation tissue response, and infection due to its crystallinity and hydrophobicity. PLLA or PDLA is resistant to hydrolysis. Thus, bioresorption with complete loss of strength in vitro does not occur within the first two years of implantation [3].

Copolymers of polyglycolic acid (PGA), PLLA, and PDLA were developed in the 1990s as the second generation, rapidly bioresorbable osteosynthetic materials are preferred over the first generation sole PLLA [4]. The materials of the second generation improved the aforementioned critical problems. This copolymer is structured to provide adequate strength for 6–8 weeks, a complete resorption time of 12–18 months, and feasibility in clinical applications for midfacial osteosynthesis as secure and rapidly bioresorbable materials [5]. In a clinical setting, this material has the advantage of rapid degradation within approximately one year after implantation.

**Figure 1:**
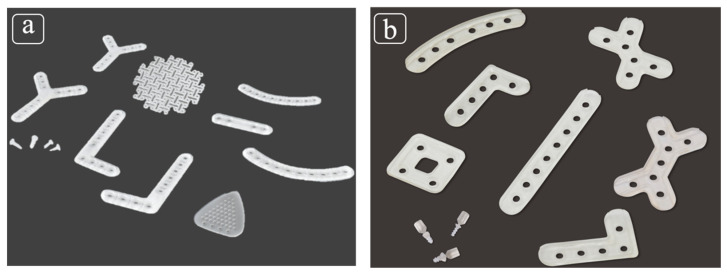
(**a**) RapidSorb^®^, a copolymer of poly-L-lactic acid (PLLA) (85%) and polyglycolic acid (PGA) (15%); (**b**) Lactosorb^®^, a copolymer of PLLA (82%) and PGA (18%).

To date, limited approval for use in clinical applications in oral and maxillofacial surgery has only been obtained by Japan Health insurance and two commercially available products of second-generation bioresorbable osteosynthetic materials in Japan, namely, RapidSorb^®^ (DePuy Synthes CMF, West Chester, PA, USA) and Lactosorb^®^ (Biomet Inc., Jacksonville, FL, USA); both products are copolymers of PLLA and PGA. The ratio of PLLA to PGA is 85:15 in RapidSorb^®^ (Figure 1a) and 82:18 in Lactosorb (Figure 1b). 

RapidSorb^®^ has the following four features: (1) because of the semi-crystalline structure called poly(L-lactide), RapidSorb^®^ breaks down without causing late inflammatory complications or foreign body reactions in vivo; (2) there is less potential migration or translocation caused by metal implants; (3) there is no need for secondary surgeries such as nail extraction; and (4) the radiation permeable polymer does not interfere with radiographs during and after surgery [6,7]. On the other hand, Lactosorb^®^ has the following five features: (1) it is resorbed into the body in about 12 months; (2) the palpability as an implant decreases with resorption; (3) there is less possibility of restriction of the patient’s bone growth and screw migration; (4) there is less incidence of inflammatory reactions and postoperative infections; and (5) there is no need for secondary surgery to remove the plate and screw [8,9,10].

These products are only ideal and approved for use only in the midface and maxillary osteosynthesis within national health insurance coverage in Japan. Our clinical research group’s recent retrospective clinical study, elucidated as a preliminary study, emphasized the feasibility of PLLA/PGA copolymer plate systems as the second generation in maxillofacial osteosynthesis with relatively small postoperative complication rates [11]. A previous report indicated 55 Le Fort I osteotomies followed up for five years to verify the development of complications, and only one patient showed mild signs of infection which was cured by antibiotics [12]. A randomized prospective study comparing titanium osteosynthesis with resorbable osteosynthesis material (Lactosorb^®^) in 60 patients undergoing Le Fort I osteotomy for long-term stability and complication rates showed no statistically significant change in maxillary position from 6 weeks to 12 months in either treatment group. Moreover, no clinically significant changes in the maxillary bone were observed in either treatment group, and all favorable treatment results were reported [13]. Therefore, the bone-resorbable plate is considered to have high clinical applicability and exceeds metallic titanium plates’ performance in terms of durability, resorbability, palpability, and the lack of need for secondary surgery. However, it caused relatively high intraoral exposure-related complications when applied to the sites close to the dentoalveolar oral regions or when a thicker plate system was used while using the Lactosorb^®^ system [13]. Norholt et al. followed postoperative complications of titanium and bioresorbable plates over one year and reported two cases of infection and wound dehiscence in the Lactosorb group, while titanium osteosynthesis was palpable in three cases after 6 to 12 months and required surgical removal [13]. This system is relatively thick and wide in order to provide sufficient stiffness for reinforcement; however, the resulting increase in the mass increases the risk of exposure.

Therefore, a review of recent studies shows that bioresorbable plates tend to be more beneficial than metallic titanium plates. However, if we aim to improve bioresorbable plates’ clinical performance, we can find further issues by showing the clinical performance of currently available bioresorbable plate systems and comparing bioresorbable plates’ clinical performance. However, a detailed long-term study has not been conducted, especially in the field of oral and maxillofacial surgery. Furthermore, comparative clinical research has not been conducted to reveal the feasibility and applicability of the two second-generation PLLA/PGA copolymer bioresorbable plate systems (RapidSorb^®^ and Lactosorb^®^) for midfacial osteosynthesis.

Therefore, the purpose of this retrospective study was to clinically evaluate and examine the incidence of postoperative complications while comparing the two PLLA/PGA copolymer bioresorbable plate systems (RapidSorb^®^ and Lactosorb^®^) in maxillofacial surgical applications.

## 2. Materials and Methods

This retrospective cohort study was designed based on a previous study by Sukegawa et al., including patient enrollment criteria and complication criteria as the primary outcome [11].

### 2.1. Patients

The retrospective clinical research data were obtained from the Department of Oral and Maxillofacial Surgery at Shimane University Hospital from 2012 to 2019. We included all patients who underwent the placement of PLLA/PGA acid copolymer plate systems (RapidSorb^®^; Synthes, West Chester, PA, USA, and LactoSorb^®^; Lorenz Surgical, Jacksonville, FL, USA) in maxillofacial surgeries of the midfacial region and had more than six months of regularly documented clinical postoperative follow-ups. This study was conducted with the approval of the Medical Ethics Committee of Shimane University (No. 4115).

#### 2.1.1. Inclusion Criteria

The patients who underwent maxillofacial surgeries of the midfacial region, namely, midfacial fracture/trauma or maxillary osteotomy for dentofacial deformity surgery using the bioresorbable materials of PLLA/PGA copolymer plate systems (RapidSorb^®^ and Lactosorb^®^) between 2012 and 2019 at the Department of Oral and Maxillofacial Surgery, Shimane University Hospital, Shimane, Japan. 

All surgical procedures were performed by two expert maxillofacial surgeons (T.K. and M.K.) who were experienced in handling these bioresorbable plate systems in a single institute.

The patients had more than six months of regular clinical postoperative follow-ups documented in our clinical and radiographic evaluation medical record charts, at one week and 1, 3, 6, and 12 months. They were followed for up to 12 months when osteosynthesis was insufficient for six months [11].

#### 2.1.2. Exclusion Criteria

We excluded patients who did not meet the inclusion criteria and did not regularly attend follow-up evaluations for up to six months [11].

We excluded patients with a compromised disease, such as uncontrolled diabetes, long-term steroid use, and patients with a severe bone disease using bone resorption inhibitory agents for bone metastasis or osteoporosis.

### 2.2. Data Collection

An evaluation grid was created to obtain all pertinent detailed patient information (e.g., age, sex, Brinkman index, medical history, clinical diagnosis of midfacial fracture/trauma or maxillary osteotomy for dentofacial deformity surgery, type of surgery for internal fixation, location of the plate placement, type of plate system, and plate selection), and correctly document any abnormal postoperative clinical events. Patients were followed up postoperatively, and the functional status and treatment complications (including infection, swelling, osseo-skeletal nonunion or malunion, plate exposure or plate breakage, plate removal, and reoperation) were assessed through clinical and radiographic examinations using computed tomography (CT) or plain radiography at 1, 3, 6, and 12 months postoperatively [11].

### 2.3. Study Outcomes

**Figure 2:**
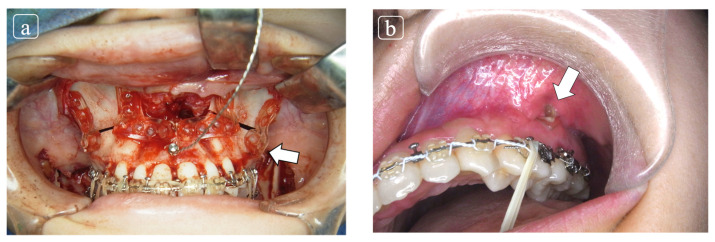
(**a**) The patient underwent orthogenetic surgery comprised by Le Fort I osteotomy for maxillary adjustment with internal fixation using Lactosorb^®^ and bilateral saggital split ramus osteotomy. (**b**) At 2 months follow-up at our outpatient clinic, the plate exposure was elucidated at infra zygomatic crest. The conservative treatment was planned and complete closure was obtained.

Clinical complications include infection, swelling, osseo-skeletal nonunion or malunion, plate exposure or plate breakage, plate removal, and reoperation (Figure 2a,b) [11].

### 2.4. Statistical Analysis

Since none of the patients had missing data, imputation was not conducted. In descriptive statistics, the median (interquartile range (IQR)) was calculated. The Chi-squared and Mann–Whitney U tests were used to compare the two groups (complication or not). A multivariable logistic regression analysis was used to predict complications based on confounding covariates, including sex, age, diagnosis, medical history, Brinkman index, plate system, plate thickness, and the number of plates. Finally, multivariable logistic regressions were also performed to adjust for group differences using the propensity score to create stabilized weights, defined as the inverse probability of treatment weighting (IPTW) [14,15,16]. All statistical analyses were performed using SPSS version 27.0 software (IBM Japan, Tokyo, Japan). Statistical significance was set at *p* < 0.05.

## 3. Results

### 3.1. Patient Characteristics

A total of 148 participants (RapidSorb^®^: 61; LactoSorb^®^: 87) who underwent maxillofacial surgery were enrolled in this study. The patients were male (81 (54.7%)) and females (67 (45.3%)). The median (IQR) Brinkman index was 0.0 (0.0–0.0). The medical history included type 2 diabetes mellitus in eight patients (5.4%), osteoporosis in six patients (4.1%), and steroid in three patients (2.0%). The diagnosis was midfacial fracture/trauma in 101 patients (68.2%) and dentofacial deformity in 47 patients (31.8%). The types of surgery for internal fixation were Le Fort I osteotomy in 41 patients (27.7%), maxillary anterior osteotomy in six patients (4.1%), maxillary fracture in 24 patients (16.2%), zygomatic arch/zygomatic fracture in 38 patients (25.7%), multiple midfacial fractures in 30 patients (20.3%), orbital floor fracture in four patients (2.7%), naso-orbito-ethmoidal fracture in three patients (2.0%), and frontal bone fracture in two patients (1.4%). The median (IQR) number of plates was 2.5 (2.0–4.0). The plates’ thickness was 1.5 mm in 45 patients (30.4%) and 2.0 mm in 103 patients (69.6%). The site of the plate placement was pyriform aperture in 75 (50.7%), periorbita in 58 (39.2%), orbital floor in three (2.0%), alveolar bone in 11 (7.4%), maxillary sinus anterior wall in 12 (8.1%), frontal bone in two (1.4%), infra zygomatic crest in 96 (64.9%), and zygomatic arch in 13 (8.8%) patients. The detailed patient characteristics are summarized in Table 1.

### 3.2. Scheme of the Plate Placement Site and the Number of Plates at Each Site

**Figure 3:**
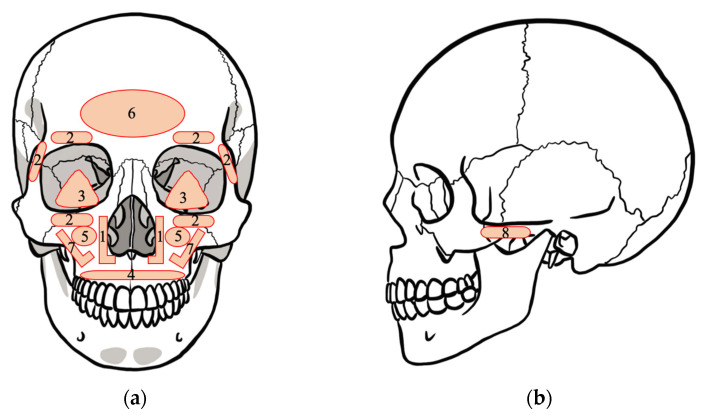
Schema of the position where the plate is placed: (**a**) coronal section of skull; (**b**) sagittal section of skull. The label numbers on the skull refer to: (1) pyriform aperture, (2) periorbita, (3) orbital floor, (4) alveolar bone, (5) maxillary sinus anterior wall, (6) frontal bone, (7) infra zygomatic crest, and (8) zygomatic arch.

The plates were placed at the pyriform aperture (127 plates), periorbita (79 plates), orbital floor (3 plates), alveolar bone (12 plates), maxillary sinus anterior wall (14 plates), frontal bone (2 plates), infra zygomatic crest (135 plates), and zygomatic arch (14 plates) (Figure 3). The total number of plates was 386.

### 3.3. Comparison of Background Factors between Non-Complication and Complication Group

Patients were divided into non-complication and complication groups retrospectively. The variables are summarized for each group in Table 2. The comparison between the two groups revealed significant differences in sex (*p* < 0.01), diagnosis (*p* < 0.01), and periorbital plate placement (*p* < 0.01). Additionally, the complication rate for RapidSorb^®^ was 4.9%, and for Lactosorb^®^ it was 14.9%.

### 3.4. Univariate Analysis, Multivariate Analysis, and Propensity Score-Adjusted Model

We performed multivariate logistic regression analysis and the propensity score-adjusted model (stabilized inverse probability of treatment weighting: IPTW) to identify factors influencing the complication rate (Table 3). There was a significant correlation of sex (OR, 10.43; 95% CI (2.28,47.80)), diagnosis (OR, 4.28 95% CI (1.45,12.61)), and periorbita (OR, 0.09; 95% CI (0.01,0.68)) in univariate logistic regression analysis. There was a significant correlation between sex (OR, 14.27; 95% CI (2.42,84.27)), plate system (OR, 5.58; 95% CI (1.27,24.45)), number of plates (OR, 4.17; 95% CI (1.26,13.82)), pyriform aperture (OR, 0.02; 95% CI (0.001,0.43)), and periorbita (OR, 0.006; 95% CI (0.0002,0.173)) in multivariate logistic regression analysis. Additionally, the propensity score-adjusted model showed a significant difference in plate systems (OR, 1.007; 95% CI (1.001,1.055)).

## 4. Discussion

Bioresorbable materials are increasingly used for bone fixation because of the benefits of accelerated bone healing and reduced risk of postoperative complications, resulting in a lower incidence of repeated maxillofacial surgeries [11]. Complication rates of 6.38–7.82% have been previously reported for bioresorbable materials [17,18]. Another study observed infection in two of 59 patients (3.4%) who received biodegradable fixation systems of plates and screws to stabilize facial fractures [19]. The complication rate we encountered with RapidSorb^®^ (4.9%) was similar to that reported previously for titanium plates. The complication rate of Lactosorb^®^ was higher (14.9%) than that of RapidSorb^®^. However, the confounder-adjusted model showed that the odds ratio was very low at 1.007, suggesting that the difference between the two materials may not be significant. Therefore, we concluded that our study’s complication rates were similar to those reported elsewhere among similar target populations.

We observed significant sex differences between the two comparison groups. Complications following the repair of facial fractures depend on the fracture location, injury severity, and various patient factors, such as age, sex, and alcohol and tobacco use [20,21]. A meta-analysis by Torgerson et al. reported that female hormones (estrogen and progesterone) were strongly correlated with bone metabolism and fracture and that hormone replacement therapy resulted in a statistically significant reduction in nonvertebral fractures. However, this effect may be attenuated in older women [22]. Since sex (female) was also a significant risk factor for complications, older women should be particularly monitored for complications after maxillofacial fracture surgery. Furthermore, hormone replacement therapy may decrease the incidence rate of complications in younger women with maxillofacial fractures [23]. Next, a significant difference was observed in diagnosis (midfacial fracture/trauma or Le Fort I osteotomy), which may be attributed to the fact that the most common complication, plate exposure, occurred in the Infrazygomatic crest of the Le Fort I osteotomy. Therefore, the anatomical location may affect the incidence of complications and diagnosis (midfacial fracture/trauma or Le Fort I osteotomy)-based differences seem unlikely. The incidence of complications in the periorbita was low. We only found one case report of complications using a bioabsorbable plate for periorbital fractures [24]. Thus our results suggest that periorbital fixation with a bioresorbable plate may be almost free of complications.

In multivariate logistic regression analysis adjusted for confounders, the plate system (Lactosorb^®^), sex (female), number of plates, pyriform aperture, and periorbita were significantly correlated with complications for both PLLA/PGA copolymer plate systems. The higher odds ratio for being female could be explained by the role of female hormones, as already discussed. However, we were unable to locate any previous research reporting that the number of plates is a risk factor for complication in open reduction and internal plate fixation (ORIF) in the maxillofacial region. Review articles in the field of orthopedic surgery suggest that the incidence rate of complications increases with the number of plates used in ORIF [25]. Hence, it seems likely that in the maxillofacial region as well, the greater the number of plates, the more likely that complications may occur. Moreover, our multivariate analysis results confirmed previous reports that plate placement at the pyriform aperture and periorbital location carried decreased risk [26,27,28,29]. Moreover, Kenneth et al. reported that 16% of patients in the transoral group had post-treatment complications compared to 10% in the transbuccal group [30]. Therefore, we concluded that the relationship between plate placement position, such as at the infra-zygomatic crest and the surgical approach related to the oral cavity, is the most important factor in complications, particularly infection by oral microorganisms. Additionally, surgical wounds near the plate, such as in Le Fort I osteotomy and fracture, are correlated with a high prevalence of wound complications after open reduction and internal plate fixation in orthopedic patients [31]. Particular attention should be paid to the possibility of plate exposure if a bulky resorbable plate system is used. Sukegawa et al. reported that plate thickness influenced the complication of plate exposure [11]. However, plate exposure mostly healed with conservative treatment in our patients, and plate removal was not needed. This problem could be solved by considering the surgical incision line’s location, taking the distance from the plate and the plate profile into consideration, and ensuring that the screw head has reduced thickness. Therefore, we recommend that bioresorbable plate systems be applied to maxillofacial surgery, but attention should be paid to risk factors, namely, the use of transoral approach and the surgical incision line site considering the infra-zygomatic crest at Le Fort I dentofacial deformity surgery.

In an analysis adjusted for confounders using the IPTW method, the use of Lactosorb^®^ was identified as a significant risk factor, but the odds ratio was only 1.007, suggesting comparable performance in clinical practice. We consider this result explained by the difference in mechanical shape and not the plate composition [32]. Sukegawa et al. reported that the removed LactoSorb^®^ plates’ molecular weight in cases without exposure-related complications was markedly reduced, confirming this plate system’s rapid and favorable resorption by the living human body [11,32]. Therefore, we predict that RapidSorb^®^ would have similar bioresorbable properties. Furthermore, we analyzed thickness as a risk factor for complications; however, there was no correlation between major complications and material-related factors. 

To the best of our knowledge, this is the first study in the world to identify comprehensive complication rates and risk factors for two types of second-generation bioresorbable maxillofacial/midfacial osteosynthetic plate systems. A recent meta-analysis discussed two trials comparing the complications of bioresorbable and conventional titanium fixation in Le Fort I dentofacial deformity surgery and three other trials comparing the complications of absorbable and titanium fixation in the bimaxillary Le Fort I plus bilateral sagittal split ramus osteotomy [5]. They found no significant difference between the two groups in terms of complication rates [5]. However, the titanium plate requires subsequent removal to prevent complications. Timing for removal of the plate has also been reviewed: most plates were removed within six months to one year of plate placement [20,33,34,35]. Moreover, surgeons insisted that the plates had to be removed in nearly all internal fixation cases, regardless of the anatomic location [36]. Bioresorbable plates have the advantage that the plate does not need to be removed, thus avoiding another surgery. The indications and use of bioabsorbable plates in maxillofacial surgery are expected to expand further.

Further development and improvement of these second-generation bioresorbable materials for use as maxillofacial osteosynthetic plate systems should reduce foreign-body reactions and enhance biocompatibility, mechanical strength, bioactivity, or bioresorption rate control, and customizability. Overall improvements in the field of oral and maxillofacial surgery and maxillofacial osteosynthesis are needed.

Our study has some limitations. The sample size was limited, and it was a single-center retrospective study design, which may have biased the results. Additionally, there was plate selection bias. Lastly, the long-term prognosis could not be evaluated due to the limited follow-up period.

## 5. Conclusions

RapidSorb^®^ and LactoSorb^®^, the two PLLA/PGA copolymer plate systems commercially available for clinical use in Japan, showed a low incidence of complications. Our data suggested that plate integration and survivability were similar when using 2.0-mm or 1.5-mm bioresorbable plates. There was a significant correlation between the infra-zygomatic crest plate placement and complications when using PLLA/PGA copolymer plate systems. Our findings suggest that female sex and a greater number of plates are risk factors for postoperative complications; pyriform aperture and periorbital as plate placement sites reduce the risk of complications. Bioresorbable plate systems can be applied to maxillofacial surgery; however, careful consideration should be taken when using a transoral approach and placing plates at the infra-zygomatic crest in Le Fort I osteotomies for dentofacial deformity surgery.

## Figures and Tables

**Table 1:** Demographic characteristics of participants (*n* = 148).

Characteristics		*n* (%) or Median (IQR)		
		Total (*n* = 148)	RapidSorb^®^ (*n* = 61)	Lactosorb^®^ (*n* = 87)
Age		37.5 (21.3–72.0)	42.0 (24.5–74.0)	34.0 (18.0–68.0)
Sex	Male	81 (54.7)	35 (57.4)	46 (52.9)
	Female	67 (45.3)	26 (42.6)	41 (47.1)
Brinkman index		0.0 (0.0–0.0)	0.0 (0.0–0.0)	0.0 (0.0–0.0)
Type 2 diabetes mellitus (yes)		8 (5.4)	5 (8.2)	3 (3.4)
Osteoporosis (yes)		6 (4.1)	2 (3.3)	4 (4.6)
Steroid (yes)		3 (2.0)	1 (1.6)	2 (2.3)
Diagnosis	Midfacial fracture/trauma	101 (68.2)	42 (68.9)	59 (67.8)
	Dentofacial deformity	47 (31.8)	19 (31.1)	28 (32.2)
Type of surgery for internal fixation	Le Fort I osteotomy	41 (27.7)	16 (26.2)	25 (28.7)
	Maxillary anterior osteotomy	6 (4.1)	3 (4.9)	3 (3.4)
	Maxillary fracture	24 (16.2)	7 (11.5)	17 (19.5)
	Zygomatic arch/zygomatic fracture	38 (25.7)	20 (32.8)	18 (20.7)
	Multiple midfacial fracture	30 (20.3)	13 (21.3)	17 (19.5)
	Orbital floor fracture	4 (2.7)	2 (3.3)	2 (2.3)
	Naso-orbito-ethmoidal fracture	3 (2.0)	0 (0.0)	3 (3.4)
	Frontal bone fracture	2 (1.4)	0 (0.0)	2 (2.3)
Number of plates		2.5 (2.0–4.0)	3.0 (2.0–4.0)	2.0 (1.0–4.0)
Thickness of plates	1.5 mm	45 (30.4)	11 (18.0)	34 (39.1)
	2.0 mm	103 (69.6)	50 (82.0)	53 (60.9)
Site of the plate placement (yes)	Pyriform aperture	75 (50.7)	30 (49.2)	45 (51.7)
	Periorbita	58 (39.2)	30 (49.2)	28 (32.2)
	Orbital floor	3 (2.0)	0 (0.0)	3 (3.4)
	Alveolar bone	11 (7.4)	5 (8.2)	6 (6.9)
	Maxillary sinus anterior wall	12 (8.1)	5 (8.2)	7 (8.0)
	Frontal bone	2 (1.4)	0 (0.0)	2 (2.3)
	Infra zygomatic crest	96 (64.9)	44 (72.1)	52 (59.8)
	Zygomatic arch	13 (8.8)	6 (9.8)	7 (8.0)

IQR, interquartile range.

**Table 2:** Comparison of background factors between non-complication and complication groups (*n* = 148).

Characteristics		*n* (%) or Median (Interquartile Range: IQR)		
		Non-Complication (*n* = 132)	Complication (*n* = 16)	*p*-Value
Age		40.0 (22.0–74.0)	27.5 (17.3–46.0)	0.08 ^a^
Sex	Male	79 (59.8)	2 (12.5)	<0.01 *^b^
	Female	53 (40.2)	14 (87.5)	
Brinkman index		0.0 (0.0–0.0)	0.0 (0.0–0.0)	0.60 ^a^
Type 2 diabetes mellitus (yes)		8 (6.1)	0 (0.0)	0.60 ^b^
Osteoporosis (yes)		6 (4.5)	0 (0.0)	1.00 ^b^
Steroid (yes)		3 (2.3)	0 (0.0)	1.00 ^b^
Diagnosis	Midfacial fracture/trauma	95 (72.0)	6 (37.5)	<0.01 *^b^
	Dentofacial deformity	37 (28.0)	10 (62.5)	
Type of surgery for internal fixation	Le Fort I osteotomy	31 (23.5)	10 (62.5)	-
	Maxillary anterior osteotomy	6 (4.5)	4 (25.0)	
	Maxillary fracture	20 (15.2)	0 (0.0)	
	Zygomatic arch/zygomatic fracture	38 (28.8)	0 (0.0)	
	Multiple midfacial fracture	28 (21.2)	2 (12.5)	
	Orbital floor fracture	4 (3.0)	0 (0.0)	
	Naso-orbito-ethmoidal fracture	3 (2.3)	0 (0.0)	
	Frontal bone fracture	2 (1.5)	0 (0.0)	
Plate system	RapidSorb^®^	58 (43.9)	3 (18.8)	0.06 ^b^
	Lactosorb^®^	74 (56.1)	13 (81.3)	
Number of plates		2.0 (2.0–3.8)	4.0 (2.0–4.0)	0.05 ^a^
Thickness of plates	1.5 mm	43 (32.6)	2 (12.5)	0.15 ^b^
	2.0 mm	89 (67.4)	14 (87.5)	
Site of the plate placement	Pyriform aperture	65 (49.2)	10 (62.5)	0.43 ^b^
	Periorbita	57 (43.2)	1 (6.3)	<0.01 *^b^
	Orbital floor	3 (2.3)	0 (0.0)	1.00 ^b^
	Alveolar bone	9 (6.8)	2 (12.5)	0.34 ^b^
	Maxillary sinus anterior wall	11 (8.3)	1 (6.3)	1.00 ^b^
	Frontal bone	2 (1.5)	0 (0.0)	1.00 ^b^
	Infra zygomatic crest	83 (62.9)	13 (81.3)	0.18 ^b^
	Zygomatic arch	12 (9.1)	1 (6.3)	1.00 ^b^
Type of complication	Plate exposure	-	11 (68.8)	-
	Infection	-	4 (25.0)	-
	Plate breakage	-	1 (6.3)	-
Complication site	Pyriform aperture	-	2 (12.5)	-
	Alveolar bone	-	2 (12.5)	-
	Maxillary sinus anterior wall	-	1 (6.3)	-
	Infra zygomatic crest	-	11 (68.8)	-
Complication date (day)		-	81.5 (43.8–128.8)	-

IQR: interquartile range; ^a^ Mann–Whitney U test; ^b^ Chi-squared test. *: significant difference (*p* < 0.05).

**Table 3:** Multivariate logistic regression analysis and propensity score-adjusted model (stabilized inverse probability of treatment weighting (IPTW)) to identify factors influencing the complication rate (*n* = 148).

Characteristics	Univariate Analysis		Multivariate Analysis	
	OR (95% CI)	*p*-Value	OR (95% CI)	*p*-Value
Age	0.98 (0.95–1.00)	0.06		
Sex (female)	10.43 (2.28–47.80)	<0.01 *	14.27 (2.42–84.27)	<0.01 *
Diagnosis (dentofacial deformity)	4.28 (1.45–12.61)	<0.01 *		
Type 2 diabetes mellitus (yes)	0.00 (0.00)	1.00		
Osteoporosis (yes)	0.00 (0.00)	1.00		
Steroid (yes)	0.00 (0.00)	1.00		
Brinkman index	1.00 (0.99–1.00)	0.45		
Plate system (Lactosorb^®^)	3.40 (0.92–12.48)	0.07	5.58 (1.27–24.45)	0.02 *
Thickness of plate (2.0 mm)	3.38 (0.74–15.55)	0.12		
Number of plates	1.47 (0.96–2.25)	0.08	4.17 (1.26–13.82)	0.02 *
Pyriform aperture (yes)	1.72 (0.59–5.00)	0.32	0.02 (0.001–0.43)	0.01 *
Periorbita (yes)	0.09 (0.01–0.68)	0.02 *	0.006 (0.0002–0.173)	<0.01 *
Orbital floor (yes)	0.00 (0.00)	1.00		
Alveolar bone (yes)	1.95 (0.38–9.95)	0.42		
Maxillary sinus anterior wall (yes)	0.73 (0.09–6.09)	0.77		
Frontal bone (yes)	0.00 (0.00)	1.00		
Infra zygomatic crest (yes)	2.56 (0.69–9.43)	0.16		
Zygomatic arch (yes)	0.67 (0.08–5.50)	0.71		
Propensity score-adjusted model (stabilized IPTW)				
Plate system (Lactosorb^®^)	1.007 (1.001–1.055)	<0.01 *		

OR: odds ratio, CI: confident interval. *: significant difference (*p* < 0.05).

## References

[1] Kanno T., Sukegawa S., Furuki Y., Nariai Y., Sekine J. (2018). Overview of innovative advances in bioresorbable plate systems for oral and maxillofacial surgery. Jpn. Dent. Sci. Rev..

[2] Pihlajamaki H., Bostman O., Hirvensalo E., Tormala P., Rokkanen P. (1992). Absorbable pins of self-reinforced poly-L-lactic acid for fixation of fractures and osteotomies. J. Bone Jt. Surg. Br..

[3] Yolcu U., Alan H., Malkoc S., Bozkurt S.B., Hakki S.S. (2015). Cytotoxicity evaluation of bioresorbable fixation screws on human gingival fibroblasts and mouse osteoblasts by real-Time Cell Analysis. J. Oral Maxillofac. Surg..

[4] Schumann P., Lindhorst D., Wagner M.E., Schramm A., Gellrich N.C., Rucker M. (2013). Perspectives on resorbable osteosynthesis materials in craniomaxillofacial surgery. Pathobiology.

[5] Yang L., Xu M., Jin X., Xu J., Lu J., Zhang C., Tian T., Teng L. (2013). Complications of absorbable fixation in maxillofacial surgery: A meta-analysis. PLoS ONE.

[6] Bergsma J.E., de Bruijn W.C., Rozema F.R., Bos R.R., Boering G. (1995). Late degradation tissue response to poly(L-lactide) bone plates and screws. Biomaterials.

[7] Bostman O.M., Pihlajamaki H.K. (1998). Late foreign-body reaction to an intraosseous bioabsorbable polylactic acid screw. A case report. J. Bone Jt. Surg. Am..

[8] Eppley B.L., Reilly M. (1997). Degradation characteristics of PLLA-PGA bone fixation devices. J. Craniofac. Surg..

[9] Pietrzak W.S., Eppley B.L. (2006). Stability of craniofacial PLLA/PGA copolymer bioabsorbable screws. J. Craniofac. Surg..

[10] Goldstein J.A., Quereshy F.A., Cohen A.R. (1997). Early experience with biodegradable fixation for congenital pediatric craniofacial surgery. J. Craniofac. Surg..

[11] Sukegawa S., Kanno T., Matsumoto K., Sukegawa-Takahashi Y., Masui M., Furuki Y. (2018). Complications of a poly-L-lactic acid and polyglycolic acid osteosynthesis device for internal fixation in maxillofacial surgery. Odontology.

[12] Toro C., Robiony M., Zerman N., Politi M. (2005). Resorbable plates in maxillary fixation. A 5-year experience. Minerva Stomatol..

[13] Norholt S.E., Pedersen T.K., Jensen J. (2004). Le Fort I miniplate osteosynthesis: A randomized, prospective study comparing resorbable PLLA/PGA with titanium. Int. J. Oral Maxillofac. Surg..

[14] Zhu J., Sharma D.B., Gray S.W., Chen A.B., Weeks J.C., Schrag D. (2012). Carboplatin and paclitaxel with vs without bevacizumab in older patients with advanced non-small cell lung cancer. JAMA.

[15] Lunceford J.K., Davidian M. (2004). Stratification and weighting via the propensity score in estimation of causal treatment effects: A comparative study. Stat. Med..

[16] Robins J.M., Hernan M.A., Brumback B. (2000). Marginal structural models and causal inference in epidemiology. Epidemiology.

[17] Kelley P., Crawford M., Higuera S., Hollier L.H. (2005). Two hundred ninety-four consecutive facial fractures in an urban trauma center: Lessons learned. Plast. Reconstr. Surg..

[18] Kirkpatrick D., Gandhi R., Van Sickels J.E. (2003). Infections associated with locking reconstruction plates: A retrospective review. J. Oral Maxillofac. Surg..

[19] Bell R.B., Kindsfater C.S. (2006). The use of biodegradable plates and screws to stabilize facial fractures. J. Oral Maxillofac. Surg..

[20] Murthy A.S., Lehman J.A. (2005). Symptomatic plate removal in maxillofacial trauma: A review of 76 cases. Ann. Plast. Surg..

[21] Furr A.M., Schweinfurth J.M., May W.L. (2006). Factors associated with long-term complications after repair of mandibular fractures. Laryngoscope.

[22] Torgerson D.J., Bell-Syer S.E. (2001). Hormone replacement therapy and prevention of nonvertebral fractures: A meta-analysis of randomized trials. JAMA.

[23] Szabo A., Janovszky A., Pocs L., Boros M. (2017). The periosteal microcirculation in health and disease: An update on clinical significance. Microvasc. Res..

[24] Doh G., Bahk S., Hong K.Y., Lim S., Han K.M., Eo S. (2018). Delayed formation of sterile abscess after zygomaticomaxillary complex fracture treatment with bioabsorbable plates. Arch. Craniofac. Surg..

[25] Wijdicks F.J., Van der Meijden O.A., Millett P.J., Verleisdonk E.J., Houwert R.M. (2012). Systematic review of the complications of plate fixation of clavicle fractures. Arch. Orthop. Trauma Surg..

[26] Francel T.J., Birely B.C., Ringelman P.R., Manson P.N. (1992). The fate of plates and screws after facial fracture reconstruction. Plast. Reconstr. Surg..

[27] Stone I.E., Dodson T.B., Bays R.A. (1993). Risk factors for infection following operative treatment of mandibular fractures: A multivariate analysis. Plast. Reconstr. Surg..

[28] Nagase D.Y., Courtemanche D.J., Peters D.A. (2005). Plate removal in traumatic facial fractures: 13-year practice review. Ann. Plast. Surg..

[29] O'Connell J., Murphy C., Ikeagwuani O., Adley C., Kearns G. (2009). The fate of titanium miniplates and screws used in maxillofacial surgery: A 10 year retrospective study. Int. J. Oral Maxillofac. Surg..

[30] Wan K., Williamson R.A., Gebauer D., Hird K. (2012). Open reduction and internal fixation of mandibular angle fractures: Does the transbuccal technique produce fewer complications after treatment than the transoral technique?. J. Oral Maxillofac. Surg..

[31] Ding L., He Z., Xiao H., Chai L., Xue F. (2013). Risk factors for postoperative wound complications of calcaneal fractures following plate fixation. Foot Ankle Int..

[32] Sukegawa S., Kanno T., Nagano D., Shibata A., Sukegawa-Takahashi Y., Furuki Y. (2016). The clinical feasibility of newly developed thin flat-type bioresorbable osteosynthesis devices for the internal fixation of zygomatic fractures: Is there a difference in healing between bioresorbable materials and titanium osteosynthesis?. J. Craniofac. Surg..

[33] Islamoglu K., Coskunfirat O.K., Tetik G., Ozgentas H.E. (2002). Complications and removal rates of miniplates and screws used for maxillofacial fractures. Ann. Plast. Surg..

[34] Rallis G., Mourouzis C., Papakosta V., Papanastasiou G., Zachariades N. (2006). Reasons for miniplate removal following maxillofacial trauma: A 4-year study. J. Craniomaxillofac. Surg..

[35] Bhatt V., Chhabra P., Dover M.S. (2005). Removal of miniplates in maxillofacial surgery: A follow-up study. J. Oral Maxillofac. Surg..

[36] Hernandez Rosa J., Villanueva N.L., Sanati-Mehrizy P., Factor S.H., Taub P.J. (2016). Review of maxillofacial hardware complications and indications for salvage. Craniomaxillofac. Trauma Reconstr..

